# Dr. Ashby Smith's Edition of the Miscellaneous Works of Dr. Willan

**Published:** 1822-06-01

**Authors:** 


*Miscellaneous Works of the late Robert Willan, M. D. F.R.S. F.A.S. comprising an Inquiry into the Antiquity of the Small Pox, Measles, and Scarlet Fever, now first published: Reports on the Diseases in London, a new edition: and detached Papers on Medical Subjects, collected from various Periodical Publications. Edited by* Ashby Smith, M. D. Licentiate of the Royal College of Physicians in London; Member of the Medical and Chirurgical Society of London, and of the Royal Medical Society of Edinburgh. One vol. 8vo, pp. 488. London, 1821.

Dr. Willan was a learned, able, and indefatigable physician, who reflected honour on his profession, and did service to his country. Dr. Ashby Smith, his "relative and friend," a young physician of promise, has piously gathered together the *disjecta membra* of Dr. Willan's productions, and added to them a hitherto unpublished memoir on the antiquity of small pox, measles, and scarlet fever, enriched with some notes and references by himself. It is highly proper that the scattered writings of eminent men should be collected by themselves or others, and left as imperishable memorials of talent well applied, for the encouragement as well as the improvement of the rising generation. Of the unpublished memoir, Dr. Smith has drawn up, in the preface to the volume, a very excellent analytical view, of which we shall take the liberty to avail ourselves in the article which we here dedicate to the work before us.

As the interest we feel in tracing the origin and progress of diseases corresponds, in some degree, to the extensive range of their visitations, and the devastations which have marked their course, it is not to be wondered at that the class of contagious eruptive fevers should have received a large portion of attention. More than a thousand writers may be cited on small-pox alone! The origin of this dire affliction is involved in obscurity, and, on that account, has been productive of great controversy. We need only allude to the discussions between De Hahn and Werlhoff in the early part of the last century, and the more recent investigations of Woodville, Moore, Monro, &c. &c.

By one class of writers it is maintained that the small-pox and measles (which were long considered to be varieties of the same disease) were known to the practitioners of Greece and Rome—by another class it is contended that their origin cannot be traced farther back than the commencement of the Mahometan æra, when the Saracens had the honour of first describing them. The *former* class assert that the descriptions extant in ancient writings resemble those of small-pox and measles as nearly as might be expected, when we take into consideration the influence of climate, customs, and habits of life—not to mention the changes which time may have produced in the character of these as of many other diseases. Another argument used on this side of the question is, that the earlier oriental writers who describe these diseases unequivocally, make no pretence to discoveries, nor to the recent introduction of such maladies—"whereas, had such been the case, it is not to be believed that they would have failed to record with their ravages, the age and birth-place of pestilences so destructive to the human race."

"The more general opinion, that the contagious eruptive fevers did not prevail among the antients, is founded almost solely upon the want of direct and precise evidence of that nature in their works; it being considered incredible that such formidable maladies, if they had indeed existed, could have escaped the attention and accurate delineation of observers, the fidelity of whose descriptions in reference to affections of far less moment, is easily recognized at the present day." *Pref.* ix.

To this it has been replied, that the paucity of information and direct testimony may be accounted for by the practice usual among the ancient physicians of referring to the same pestilential constitution, different malignant fevers—the eruptions being regarded as crises varying less in their nature than in the accidental combinations of the peccant humours. To this may be added, the dread of contagious and fatal disorders, and the general conviction that they were dispensations of divine vengeance on guilty nations, and consequently beyond the reach of medicine or human aid. Dr. Willan's arguments are in support of the affirmative of this question—namely, that the diseases alluded to *were known* to the ancients. One of his main objects therefore is to shew that these diseases were in existence when the authors in question flourished; but that looking on these complaints as merely species of the common pestilence, they treated of them conjointly with it, considering it unnecessary to assign to them particular denominations, or to leave precise and accurate descriptions of them as discriminated from the generic distemper on record.

Rhazes, the first writer who mentions small-pox under a specific name, supposed it to have existed as early as the second century, and that it was well known to Galen. The Greek translator of Rhazes's Treatise adverts to Galen's acquaintance with the disease as an undoubted fact. The title of this translation (περι λοιμικης) and its preface* proves that the small-pox had been known to the *antient* Greeks, under the name of Loimiké, (the loimic or pestilential disease,) and even divided into two species—the Loimiké and Eulogia, the latter term signifying the more troublesome species of the two. Haly F. Abbas observes that "the Al-gidri (variolæ) are numerous small ulcerations affecting the whole or greater part of the surface of the body, which the *antients* called Anthrakes; but which the (Syrian) Greeks and Arabians called daughters of fire." Constantinus Africanus says —"Antiqui vocant has (variolas) ignis carbones:" and the modern Greeks yet apply the terms Loimé and Loimic disease to the small-pox and measles.

The identity, or near resemblance at least, of these several denominations to those employed by the same people to describe the plague itself, and its characteristic eruptive symptoms (Loimos and Anthrakes) implies such an imagined close affinity between the things denoted by them, as proves the confusion of all these diseases, and goes far (Dr. Willan thinks) towards explaining why the descriptions of, or allusions to, the variolous eruptions, actually transmitted down to us in their writings, should have been overlooked by the moderns. Pursuing this idea, Dr. Willan institutes a strict analysis of the leading published statements on pestilence, and the results, he thinks, clearly evince that certain parts of them must, in almost every instance, refer to the small-pox—and to the small-pox only. For example, an examination of the epidemic which broke out at Alexandria, A. D. 252, and which spread with great fury for twelve or fifteen years, proves that pestilence was not one uniform disorder, but comprised several different kinds under it, distinguished by the narrators from the common Loimos.

"The histories of the next considerable plague in point of time (that which prevailed in Syria in the reign of Dioclesian) lead to the following deductions vitally material to the question at issue. 1. The mortality was not occasioned by one form of disease, but, *independently of the common Loimos*, there was, according to Eusebius, *another disorder* termed, from its fiery nature, Anthrax. 2. This Anthrax *spread over the whole bodies* of the sufferers. 3. The *eyes* were very frequently affected, producing blindness in thousands

* Written in the 10th or beginning of the 11th century. The translator says—"it seems strange that he who first organized the medical art, (Galen,) and defined what had been left indeterminate, should have but slightly noticed a disease (small-pox) to which every man is born liable."

of individuals. 4. Patients were not a *second time* attacked by the contagion. 5. It is by another author represented as an *ulceration attracting or draining out humours*, and attended with an offensive smell. (pp. 5, 6, 7.) Now it is very doubtful whether any of the several characteristics above specified will apply to the pestilential Bubo; certain that most of them will not; and equally certain that the aggragate will not correspond to the description of any other complaint except the small-pox: but that it will, with great fidelity, coincide with the most accurate accounts of the small-pox in its confluent form. Dr. Willan therefore concludes, and the conclusion seems to be irresistible, that the disease distinguished so frequently under the name of the *spreading* or *herpetic Anthrax* from the common Loimos, was the confluent small-pox." xv.

In the 2d chapter Dr. W. continues the inquiry through the writers of the first and second centuries. Philo Judæus, about the middle of the first century, describes a Loimic disease which agrees very nearly with Eusebius's account of the herpetic or spreading anthrax, and exhibits the mode of diffusion, and the circumstances of the confluent small-pox.* Herodotus, a physician of Asia Minor, describes, in the reign of Domitian, a febris loïmodes, without noticing buboes or carbuncles, but adverting to the existence of *ulcerative, anthrax-like, herpetic exanthemata on the face and the rest of the body;* "and observations present themselves in all the writers on small-pox, from Rhazes to Sydenham, similar to those made by Herodotus on these eruptions." Rufus, of Ephesus, says nothing of buboes or carbuncles in Loimos; but he states that—" besides other evil ulcers, the all-dreadful Anthrakodea may take place in the Loimos, *as well on the rest of the body as on the face and tonsils.*"

Galen has not left us any distinct history of Loimic diseases; but there are numerous scattered observations in his works respecting them—especially respecting a pestilential disease introduced into Asia Minor by the army of Lucius Varus, about the year 164, in which no mention is made of buboes or other glandular swellings; consequently Dr. Willan refers it to epidemic small-pox of the confluent kind, and also measles.

* We confess, however, that the Jewish philosopher's ætiology of this disease leaves an impression on the mind that it was not small-pox. He says—" the cloud of dust suddenly falling on men *and cattle*, produced over the whole skin a severe and intractable ulceration, &c." All these statements, too, must be taken *cum grano salis;* for even so late as the 14th century, a writer, after describing an epidemic small-pox, as Dr. Willan supposes, informs us that the Bishop having anointed a person, in this disease, with oil taken from the lamps burning at the tomb of Saint Marcellin, the pustules subsided, and the patient's skin became *more polished than it had formerly been.*

"1. When the disease was about to terminate favourably, *numerous exanthemata appeared over the whole body, which in the greater number of patients were ulcerative or pustular.*—2. The disease was occasionally attended with roughness and hoarseness of the voice.—3. A distinction is carefully made between two species of the exanthemata, which is precisely answerable to the long-recognized and universally known difference between the eruptions in small-pox and measles. (pp. 38, 45, and notes to 46.)—4. The sudden retrocession of pustules or tubercles efflorescing from within, rendered the case highly dangerous.—5. Almost all who perished, died of colliquative diarrhœa.—6. So great was the deformity produced in the persons of the sufferers by the ravages of the epidemic Anthrakes in Asia, that they were compared by the spectators to apes, rather than men." *Pref.* xix.

Dr. Willan thinks that, from a passage in Dion Cassius, (Rom. Hist. lib. 62,) there is some reason to believe that an attempt at inoculation had been made in the reign of Domitian, A. D. 92,) and revived under the Emperor Commodus. After mentioning the great pestilence, (A. D. 189,) Dion Cassius adds—"many died in another *way*, not only at Rome but over nearly the whole empire, through the practice of miscreants who, *by means of small pointed needles, communicated, for a reward, the horrid infection* so extensively, that no computation could be made of the numbers that perished."*

In the fourth chapter, Dr. Willan endeavours to trace evidence of the existence of small-pox in periods anterior to the Christian æra. He thinks that Hippocrates' account of the Anthrakes applies more strictly to small pox than it does to carbuncles. His words are—"The Anthrakes appeared at Cranon, in a very hot and rainy summer, mostly with a south wind. Ichors collected under the skin, and, being

* This is pretty analogous to the calumnies circulated against our early inoculators. In one of the first pamphlets published here on inoculation it is said—"not only physicians make havoc of mankind for the satisfaction of their judgment in physic, and increase of their experience, but every quack now may be a hireling to the Devil, and, like that banditti in Italy, be ready to do the drudgery of removing heirs, and other obstructing incumbents of many kinds, and to do this under the mask of a cure, inoculating death instead of disease, and making use of an art never before practised, in a manner not foreseen, and by the laws not yet sufficiently guarded against."—*See Woodville's History of Inoculation, p.* 126. These specimens of medical logic, indeed, have been quite equalled by the early antivaccinators themselves, in our own times. We think we may venture to predict, that medical controversies *in this style* have now ceased for ever. The profession will never again disgrace itself by permitting the introduction of JOHN BULL ribaldry in their discussions.

confined, they became hot, and excited itching. Then there arose phlyctænides, such as are caused by fire, and they seemed to burn under the skin." 53. But we confess that we can see little in this passage to support the design of the author in *antiquatising* small-pox.

From the Grecian, Syrian, Jewish, and Egyptian records our author naturally has recourse to those of Rome in its regal and republican states. Here our sole guides are, of course, the historians and poets. Among the former the terms pestis and pestilentia were used in the same extensive signification as Loimos and Loimiké by the Greeks, comprising every contagious epidemical disease. It is therefore contended that, if diseases are found recorded in Roman history under these denominations, and with epithets and characters not appertaining to the pestilential bubo, or epidemics caused by famine, but descriptive in different degrees of the confluent small-pox, we are justified in presuming that the diseases so described were, in some instances, at least, the confluent small-pox.

The intrepid Seneca—a poet and philosopher, is introduced by Dr. Willan as describing a disease, the Loimos at Thebes, bearing a striking resemblance to small-pox.

> " O dira novi facies leti!
> Gravior leto!—Piger ignavos
> Alligat artus languor, et ægro
> Rubor in vultu, *maculæque* caput
> *Sparsere leves;* tum vapor ipsam
> Corporis arcem flammeus urit
> Multoque genas sanguine tendit,
> *Oculique rigent,* et *sacer Ignis*
> Pascitur artus. Resonant aures
> Stillatque niger naris aduncæ
> Cruor, et venas rumpit hiantes.
> Intima creber viscera quassat
> Gemitus stridens, &c. &c.
>
> Senec. Œdip. Act. I.

Dr. Willan points out the coincidence between this eruptive disease and small-pox or measles. He does not think the circumstances attending these last eruptions are better described by any of the Arabians or other medical writers prior to Rhazes, than in the above extract.

The fourth chapter of this learned essay is dedicated to the purpose of shewing that small-pox was prevalent in the British Isles and on the Continent of Europe, prior to the supposed origin of the disease in Arabia, being described by literary men under the terms pusula—pustularum morbus, and morbus dysentericus cum pustulis. Gregory of

Tours states that, about the year 580, A.D. almost every district of France was occupied by a dreadful plague (Lues) in which the patients were affected with violent vomiting, fever, head-ache, and excruciating pain in the loins. This epidemic was particularly fatal to *children*. King Chilperic recovered with difficulty himself, but lost two of his sons. Austrigilda, Queen of Orleans, sank under this disease; but not until she had exacted a promise from the *king* that her two physicians should be put to death if they did not save her! The physicians were accordingly executed! We live in better times now. A queen may die, and her physicians may still hold up their heads—even "within the verge of the palaces." The following passage from the same author, must, Dr. Willan thinks, remove all doubts that the small-pox existed in France long before the Arabian æra.

"'Last year, the state of Tours was *desolated* by a severe pestilential sickness (Lue Valetudinariâ;)—such was the nature of the infirmity *(languor,)* that a person, after being seized with a violent fever, was covered all over with vesicles and small pustules (vesicis ac minutis pustulis.) The vesicles were white, hard, unyielding, and very painful. If the patient survived to their maturation, they broke, and began to discharge, when the pain was greatly increased by the adhesion of the clothes to the body.—In this malady, the medical art did not avail without the assistance of Saint Martin; for many were restored, who sought a benediction from his holy temple. Among others, the Lady of Count Eborin, while labouring under this pest, was so covered with the vesicles, that neither her hands, nor feet, nor any part of the body, remained exempt, for even her eyes were wholly closed up by them. When nearly at the point of death, she received some of the water, in which the tomb of the blessed saint had been washed at the Lord's Passover.—This having been taken as a drink, and applied to her sores, the fever abated, the discharge from the vesicles was made without pain, and she was soon after healed.'" 92.

We confess that this is a very strong document, and nearly decisive of the question.

In the British Museum there is a miscellaneous manuscript of the eighth or ninth century, partly Saxon, partly Latin, in which it is said that Saint Nicaise, Bishop of Rheims, A.D. 453, had been affected with a species of variola, and was at that time favoured with the privilege of emancipating his worshippers from the disease by means of a talismanic inscription to be suspended about their persons. "Sanctus Nicasius habuit *minutam variolam*, et rogavit dominum, &c. &c."

The Treatise concludes with a description from Adomnan, a learned Hibernian Scot, of an *eruptive epidemical* disease in Ireland, attended with *purulent ulcerations* which raged cotemporaneously with the pustular lues in France.

It is evident that Dr. Willan has not contented himself with consulting the ordinary sources of information on the subjects under investigation—the writings of medical authors. He has taken, in fact, a comprehensive view of the works of historians, poets, and ecclesiastical writers of antiquity. From these authorities he has accumulated a mass of *probable* evidence that the small-pox, measles, and scarlet fever, have existed in almost every age of the world, of which history or tradition has furnished us with any records. Such an inquiry is certainly very interesting, and only subordinate to an accurate investigation of the nature and treatment of these diseases at the present moment. Such inquiries, too, enlarge the mind, and thus invigorate the understanding—a due proportion of them is therefore to be encouraged in modern medical literature; on which account we have entered into the analysis of Dr. Willan's posthumous essay, somewhat farther than the able editor himself. The notes and references appended to this part of the volume by Dr. Smith are creditable to this gentleman's erudition; and we cannot help anticipating something important from the same pen, when time shall have matured his judgment, and experience supplied him with ample materials whereon to exercise it.

Of the "Reports on the Diseases in London," we need not speak, as they were first published in the periodicals of the day, and afterwards collected and republished in a separate volume, with additions by the author himself. In the present edition, however, there are notes appended by Dr. Smith, which tend to enrich these very valuable reports.

The detached papers on medical subjects collected in this volume, are five in number, viz. a remarkable case of abstinence—a case of obstruction of the bowels—singular termination of dropsy—observations on the use of arsenic in intermittents—and cases of Ischuria Renalis in children.

It is a common observation that we want facts or materials in medicine, on which to raise the superstructure of theory or science. But time buries facts in oblivion almost as fast as they are recorded. Who in the present day has time or inclination to toil back through the innumerable volumes that have been written, or the myriads of cases that have been put upon record by our predecessors? Very few indeed! A *retrospective* review in medicine is the greatest desideratum in this intellectual age; and we are perfectly satisfied that such a work would confer more benefit on medical science than all the other periodicals put together. It would also have unprecedented success; for the facts collected from the records of the past would be completely new to nine-tenths of medical society; and, with our now extended knowledge of pathology, would turn to excellent account. This has long been our conviction, and were it not that a delineation of what is *now* going on in the world requires the *whole* of our review, we certainly should put the suggestion into practice. We hope some others, more equal to the task, will take the hint here thrown out. The cases recorded only 30 or 40 years ago by the author, whose work now lies before us, are virtually unknown to the great mass of practitioners, and therefore we shall take some notice of them in this place.

The first was a melancholy case of fanatical abstinence, from which Danté might have drawn his too celebrated description of the wretched and emaciated Ugoline. A young man, studious and dyspeptic, commenced a course of abstinence in the year 1786, ostensibly to relieve certain uneasy sensations in his stomach, but secretly from some mistaken notions of a religious nature.

Having betaken himself to an obscure lodging, he entered on a system of diet, composed of water slightly acidulated with orange juice. After three days' fare of this kind, the craving for food, at first troublesome, subsided entirely, and he then pursued his meditations without inconvenience. He took no kind of exercise—slept little—and spent most part of the night in writing. The quantity of water used each day was about three-fourths of a pint; and two oranges served him a week. He made water in moderate quantity, clear and without sediment. He had a natural stool on the second day of this course, and none again till the fortieth day, which was the last, though he persisted in his aqueous regimen for twenty days longer. During the last ten days of this infatuated course his strength wasted rapidly, and when he found himself unable to rise from his bed, he got alarmed, and his delusive visions began to fade away. His friends, at this time, having discovered his retreat, a clergyman and physician (Dr. W.) were sent to him, and obtained his consent to try the means of resuscitation—he being now in the sixty-first day of his fast.

"He was at that time emaciated to a most astonishing degree; the muscles of the face being entirely shrunk; his cheek-bones and processus zygomatici stood prominent and distinct, affording a most ghastly appearance: his abdomen was concave, the umbilicus seeming to be retracted, from the collapsed state of the intestines; the skin and abdominal muscles were shrunk below the brim of the pelvis, and under the ribs, leaving the space vacant betwixt the ossa ilia, the lower ribs, and spine. His limbs were reduced to the greatest possible degree of tenuity; the ossa ischia, the internal trochanters, and all the processes of the bones being easily distinguishable.

"His whole appearance suggested the idea of a skeleton, prepared by drying the muscles upon it, in their natural situations.

"His eyes were not deficient in lustre, and his voice remained clear and sound, notwithstanding his general weakness." 440.

He was at first directed to drink a pint of barley water and two cups of panada daily—which, in our humble opinion, was too much, at first, for organs so long unaccustomed to nutriment. He had some feverish heat the first night, but slept better than before. On the second day he had some mutton tea; and on the fourth, a pint of milk for breakfast, and a pint of mutton broth for dinner. He had that day two formed motions, and was sick and vomited. For a few days every thing seemed to go on well; but in a week from the commencement of the new system the scene entirely changed. He began to lose his recollection, and before midnight became frantic and unmanageable. Pyrexia, tremors, delirium, and other symptoms set in, and he died at the end of a fortnight from the time he was first seen by Dr. Willan, quite emaciated, and more wasted than ever.

Many are the instances on record of long abstinence—some of them longer than this, of which Dr. Willan did not seem aware—but few of them are so well authenticated as to claim implicit credence.* It is curious that M. Ponteau, long ago, proposed a water diet for the eradication of cancer—and assures us that one person was completely cured by this plan. In others who could not be prevailed upon to persist long enough, the disease, he says, appeared to be very much mitigated. M. Ponteau therefore anticipated Dr. Lamb in his more recent proposals and practice of a similar kind. We are convinced that physicians and surgeons do not make such use of this powerful measure as they ought to do in the eradication or mitigation of formidable diseases—probably from the reluctance with which

* Haller, in his Elementa Physiologiæ, tom. vi. cites a great number of instances; one is that of a young woman, who passed three years without any kind of aliment. In general these cases of long abstinence have happened in females, who have lain in a torpid state, with little or no secretion or excretion to require aliment—in fact, in a state considerably analogous to that of hibernating animals. We would recommend to the curious reader a little work of Hoffman, "De Inedia, magnorum morborum Remedio," as containing many important hints and observations.—*Ed.*

patients are brought to such rigid abstinence. In tumours, aneurisms, and many organic diseases of internal parts much might be done by aqueous aliment, were it properly urged by the practitioner, and implicitly adopted by the patient.

---

The next case related by Dr. Willan is that of a remarkable obstruction of the bowels. A lady, fifty years of age, subject to frequent attacks of pain in the bowels, and generally constipated, was seized on the second of April, 1784, with the usual symptoms of colic, attended with almost constant vomiting. Purgatives of every description were exhibited, together with glysters of all kinds, including the smoak of tobacco, but without any effect. The abdomen was also cupped. After six days obstruction and great pain at intervals, there was still no fever, nor mark of irritation in the pulse. Ice was now applied to the abdomen, which gave great pain, and excited much commotion in the bowels, but produced no evacuation. On the 10th April she passed some flatus downwards, and this encouraged the attending physicians to try quicksilver, which was given to the amount of six ounces in two drachm doses. As it produced a sense of weight and uneasiness, she could not be prevailed upon to take any more of it. On the 17th and 18th there appeared some slight feculency in the motions which gave some ray of hope, but nothing more appeared, and the patient continued in this deplorable condition till the 1st of May, when she expired.

*Dissection.* The bowels were found amazingly distended throughout, particularly the caput coli, and containing not less than four gallons of fluid feculent matter. The whole tract of intestines was inflamed, and in many places sphacelated. The constriction was found at the lower part of the sigmoid flexure of the colon near the top of the sacrum. For about the length of an inch the intestine was so contracted that nothing could pass it. This portion of bowel was quite hard and callous. Some of the quicksilver was found above the stricture; a part of it also seemed to have been triturated with the mucus of the bowels into a black gelatinous mass. The other abdominal viscera were healthy.

Dr. Willan makes some reflections on this case. It is remarkable, he observes, first, that extensive inflammation, and even mortification, may take place from a gradual distention of the bowels, without producing heat, fever, or any of the common inflammatory symptoms. Secondly, it is interesting to know that a patient lived upwards of thirty days without any evacuation, and without any stercoraceous vomiting. Thirdly, that quicksilver taken into the stomach, in considerable quantity, and retained in the bowels for some time, does not always produce dangerous effects from its bulk or momentum. Fourthly, that constrictions in the large intestines are not attended with the same acute and violent symptoms, such as fever, sharp pain, constant vomiting, hiccupping, prostration of strength, &c. which generally soon prove fatal in contractions or spasms of the smaller bowels. This observation, he thinks, may help to distinguish the seat of the disease, and when we conclude that the obstruction is in the large intestines, he cautions us against persisting in the use of active purgatives and stimulant enemata, which often give unnecessary pain, and tend to aggravate the disorder. In such cases, he thinks, the introduction of a candle or bougie up the rectum and into the colon might lead to relief.

We lately attended a female in Bond Street, who had been ill with enteritis several days before we saw her, and who lived sixteen days without any evacuation. The fever, however, was considerable, and the decisive depletion, which we put in use, completely quelled this part of the complaint. But no evacuation could be procured by any of the usual means. She came at last to vomit up the contents of the small intestines daily, after which she was quite easy, cheerful, and comfortable, till the next day, when similar evacuations took place from the mouth. We are strongly disposed to think that life might have been eventually preserved, though with this dreadful appendage of daily vomiting up the fecal remains of the small intestines, had we not, on the 16th day, been induced to exhibit quicksilver. She died that night. We found about eight inches of the ileum, where it terminates in the colon, in a state of complete gordian knot, and glued together by the effects of adhesive inflammation, but all inflammation itself of this and other parts of the bowels was gone. We could not find the quicksilver at all. Mr. Tebbs, of Bond Street, was present at this dissection. There were extensive marks of repeated attacks of inflammation about the uterus and ovaria—but all fever and phlogosis had disappeared for many days previous to death. We understand there is a man now in London, who lives, with a complete obstruction in some part of the alimentary canal. He has daily one or more fits of fecal vomiting, after which he pursues his usual avocations in common health, and without ever having a motion by stool.

---

The last case which we shall notice in this volume, is that of a woman, 38 years of age, who having caught a severe cold, became soon afterwards affected with anasarcous swellings of the legs, which did not subside when the catarrhal symptoms disappeared. When our author first saw the patient she was universally bloated—her legs especially were swelled to an enormous size—and there seemed to be fluctuation in the abdomen. After the natural cessation of a menstrual discharge, there came suddenly on a flow of water *per vaginam*, which drained through the bed before she could get assistance, and afterwards filled a vessel that held three quarts, leaving her faint and languid. The evacuation continued in a more gradual manner for two days, when the dropsical effusions had entirely disappeared. Within ten days the water had again accumulated, and again the same evacuation as before took place, and with the same effect. She now went into the country, took the cinchona, and completely recovered. It is proper to observe that the patient took two grains of the digitalis thrice a day (a pretty large dose by the bye) for a fornight prior to the first evacuation of water, but with only trifling effect on the urinary secretion. "The sudden termination," says Dr. Willan, "of the disorder must be referred to the operation of the remedy coinciding with the state of the uterine vessels, and determination of blood to that organ at the time." It was, in fact, one of those *God-sends*, where nature does the business for us in a handsome way. From some cases, however, which we have seen, and others that we have heard of, we are inclined to doubt that this watery discharge was *uterine*. We have seen the *urinary* discharge so copious and involuntary sometimes that the patient could not be persuaded the fluid came from the bladder. In such cases the renal secretion had not the common characters of urine.

The paper on the use of arsenic in intermittents, and the cases of ischuria renalis in children, we are obliged to pass over, having already dedicated a considerable space to the volume under consideration.

